# 

**Published:** 1893-07

**Authors:** 


### BOOKS AND PAMPHLETS RECEIVED.

Colotomy. A Clinical Lecture on Diseases of the Rectum, delivered at the New York Post-Graduate Hospital, by Charles B. Kelsey, M. D., New York. (Reprint.)

The Present Status of Electrolysis in the Treatment of Urethral Strictures, with statistics of one hundred cases (third series,) by Robert Newman, M. D., New York. (Reprint.)

A Clinical Lecture on the Prevention of Idiopathic Rotary Lateral Curvatures of the Spine, by H. Augustus Wilson, M. D., Philadelphia, Pa. (Reprint.)

On the Value of Constitutional, together with Local Treatment, in cases of Chronic Periostitis, by Charles H. Merz, A. M., M. D., Sandusky, Ohio.

History of Surgery in South Carolina. Being the essay to which the prize offered by Dr. Joseph Price, of Philadelphia, was awarded by the South Carolina Medical Association, April 1893, by Edward Frost Parker, M. D., of Charleston, S. C. (Reprint.)

Acromegaly, with the Clinical Report of a Case, by Archibald Church, M. D., Chicago, Ill., and William Hessert, M. D., Chicago, Ill. (Reprint.)

The Cure of Complete Prolapse of the Rectum by Posterior Proctectomy, by John B. Roberts, M. D., Philadelphia, Pa. (Reprint.)

Senile Hypertrophied Prostate, by Branford Lewis, M. D., St. Louis, Mo. (Reprint.)

A study of two cases of Paroxysmal Sneezing with the Treatment, by William T. Cathell, M. D., Baltimore, Md. (Reprint.)

The internal treatment of Lupus Erythematosus with Phosphorus, by L. Duncan Bulkley, A. M., M. D. (Reprint.)

